# Green Environments and Healthy Aging: Analyzing the Role of Green Infrastructure in the Functional Well-Being of Seniors—A Pilot Study

**DOI:** 10.3390/ijerph22010035

**Published:** 2024-12-30

**Authors:** Andrea Ribeiro, Rodrigo Soares, Luis Barbosa, Ana Silva, Raquel Ferreira, Sara Terroso, Ana Claudia Andriolli, Ligia Torres Silva, Carlos A. Ribeiro

**Affiliations:** 1 Centro Interdisciplinar em Ciências da Saúde-CICS, ISAVE, Rua Castelo de Almourol nº 13, 4720-155 Amares, Portugalcarlos.ribeiro@labpaisagem.pt (C.A.R.); 2 CIR, Escola Superior de Saúde, Politécnico do Porto, Rua Doutor António Bernardino de Almeida 400, 4200-072 Porto, Portugal; 3 Laboratório da Paisagem (LdP), Rua da Ponte Romana, Creixomil, 4835-095 Guimarães, Portugal; sara.terroso@labpaisagem.pt (S.T.); acandriolli@gmail.com (A.C.A.); 4 CTAC—Research Centre for Territory, Environment and Construction, University of Minho, 4800-058 Guimarães, Portugal; lsilva@civil.uminho.pt

**Keywords:** environmental quality, physiotherapy, cardiorespiratory variables, active aging, green infrastructure

## Abstract

Health professionals have slowly integrated the environment and green areas into their prescriptions to connect patients with nature and outdoor activities. The World Health Organization recommends that everyone reside within 300 m of green regions to improve well-being and physical and mental health. The study aimed to explore the effects of urban and rural green areas on multiple physiological and functional variables, as well as evaluate the perception of individuals regarding the ease of use of these same spaces. Participants walked twice a week for six weeks, covering 1.6 km. Heart rate (HR), blood pressure (BP), oxygen saturation (SpO_2_), physical capacity analysis (IPAQ), risk of falls (Morse Fall Scale), Mini Mental State Examination, physical performance test (PPT), and perception of accessibility (Pedestrian Accessibility Perception Scale for adults over 65 years old) were evaluated/administered. Variables such as noise, temperature, and air quality were also measured during the outdoor activities. Twenty-four individuals divided into two groups participated in the study: group 1 (urban route) and group 2 (rural route). We found impacts on body weight (*p* = 0.021), SpO_2_ (*p* = 0.033), and Mini Mental State Examination (*p* = 0.041) scores in group 1 and SpO_2_ and PPT scores in group 2. This study highlights the importance of incorporating green infrastructure in urban planning to support healthy aging and improve accessibility for older adults, and shows that outdoor activities provide various health benefits (physical, mental, and social well-being) and that walking in urban and rural environments seems to impact the health of elderly individuals positively and should be considered in physical therapy by health professionals.

## 1. Introduction

Today’s society is influenced by global warming, pollution, and social inequalities, among others, which are unfavorable to everyone’s health. However, as we become more technologically sophisticated, more natural resources are used, generating more pollution [1]. In this way, the health approach must change and be thought of in an integrative way rather than in isolation, as has been the case to date [2]. Health professionals have slowly integrated the environment and green areas into their prescriptions to connect patients with nature and outdoor activities.

One of the most significant steps that physiotherapists can take is to identify physiotherapy as an environmentally friendly practice and emphasize technologically simple techniques, essentially using manual therapy and interpersonal relationships, among others [3]. Another relevant issue is sterile clinical environments and their separation from natural ecosystems, avoiding contamination.

A book by Nicodemo and Primavesi [4], refers to the importance of vegetation for human well-being. Vegetation has a clear physiological influence, such as reducing stress, blood pressure, and heart rate and improving other physiological indicators. Green spaces have become the most threatened by urban growth, and their decline is the major cause of environmental and social problems impacting human health and well-being [5]. It is important to highlight that functional well-being in older populations refers to the ability of older adults to maintain their physical, mental, and social capabilities, allowing them to perform daily activities and engage in meaningful interactions. This concept encompasses various dimensions, including physical health, psychological well-being, and social participation [6]. Maintaining functional well-being is crucial for older adults to lead independent and fulfilling lives. Sheng [7] considers that the presence of places such as city parks, among others, encourages socializing and quality of life. In contrast, the more urbanized areas seem to have a negative influence on the parameters mentioned above [8]. Air pollution is Europe’s largest environmental health risk, causing cardiovascular and respiratory diseases that impact health, reduce quality of life, and cause preventable deaths. Despite ongoing overall improvements in air quality, 96% of the EU’s urban population is exposed to unsafe concentrations of fine particulate matter (PM_2.5_: fraction of particulates with an aerodynamic diameter smaller than 2.5 μm) [9]. Ambient (outdoor) air pollution in both cities and rural areas was estimated to cause 4.2 million premature deaths worldwide per year in 2019. This mortality is due to exposure to fine particulate matter, which causes cardiovascular and respiratory disease and cancers [10].

According to the World Health Organization (WHO), 9 out of 10 people breathe air with high levels of pollution, and it is estimated that 7 million people die each year from cardiovascular and pulmonary diseases due to this. The WHO also estimated that in 2019, 37% of outdoor air pollution-related premature deaths were due to ischemic heart disease and stroke, 18% and 23% of deaths were due to chronic obstructive pulmonary disease and acute lower respiratory infections, respectively, and 11% of deaths were due to cancer within the respiratory tract [10]. Therefore, implementing green spaces in urban environments improves the environment, contributing to better air quality, attenuating atmospheric pollution, and promoting a physical environment that encourages physical exercise [11]. Therefore, the WHO recommends that everyone reside within 300 m of green areas to improve well-being and physical and mental health. Both environmental noise and air pollution have been linked to cardiovascular conditions that may affect cognition in older adults [12,13].

Environmental noise remains a major problem in Europe, with at least 20% of the EU population living in areas where noise levels are considered harmful to health. Most of the people affected live in urban areas. Road traffic is by far the most dominant source of environmental noise. The number of people exposed to high environmental noise levels has broadly remained stable since 2012 [14]. Chronic exposure to road traffic noise has been associated with faster cognitive decline in older adults, particularly affecting executive function [13].

Therapeutic exercise is a key part of physiotherapy treatment. Counseling outdoor activities aims to meet what is described in the literature by stimulating social interaction and consequently avoiding the social isolation so common today, especially among the elderly [15]. This activity can be held in rural or urban areas, and the reason for choosing one over the other is not clear.

Given the above, this study aimed to understand the influence of two areas, one rural and one urban, on multiple physiological and functional variables, as well as assess individuals’ perceptions regarding the ease or otherwise of using these areas. Environmental variables, such as noise and air quality, were measured to provide additional context.

## 2. Materials and Methods

A quasi-experimental study was carried out to meet the proposed objective. It took place in Guimarães, in urban and rural areas, after approval by the ISAVE ethics committee. The urban and rural areas were selected based on the characteristics of the study. The selection criteria were based on noise levels, air pollutant concentration, and green area presence. In this way, it was decided to choose a route located in the city center (with a high concentration of buildings and road traffic, and therefore a polluted environment) and a rural route located in a naturalized and green area (with a low concentration of buildings and good air quality and noise).

The area chosen for the urban route is in the historic center of Guimarães, in a very urbanized area, with high traffic flow (>800 vehicles/h at peak times) and a large influx of people, given the presence of several points of interest, both in terms of service and for tourist reasons. There are no industrial areas or quarries in the surrounding area, road traffic being a main source of noise and air pollution in this area. In terms of acoustic zoning, this is a mixed zone, where the Lden (day–evening–night noise level) must not exceed 65 dB(A) [16].

The area chosen for the rural circuit is in the village of Ronfe in the municipality of Guimarães, a rural area with houses, cultivation areas, an industrial area, and plenty of vegetation, particularly close to the banks of the Ave River, an area predominantly connected by local access roads. Road traffic and some contribution from industries are the main sources of air pollution and noise in this area.

The straight-line distance between urban and rural routes is 7 km. The percentage of green areas along the urban route (within a 500 m radius) is 13.6%, while the percentage for the rural route with the same radius is 68.7%.

### 2.1. Participants

A total of 24 healthy individuals, 3 men and 21 women, took part in this study, with an average age of 67.96 (±4.35) years, average height of 158.9 (±7.1) cm, and a body mass index (BMI) of 28 (±3.7) kg/m^2^, associated with Tempo Livre (Guimarães, Portugal).

All participants were retired and married, living in Guimarães in urban and rural areas, and were recruited by direct contact to participate in the study voluntarily.

Because this was a pilot study that we intend to inform future research, we performed it with 24 subjects.

Concerning sample size, we made the following calculations: significance level (α)—this is typically set at 0.05, which corresponds to a 95% confidence level; power (1 − β)—80% (or 0.80), meaning an 80% chance of detecting an effect if there is one; effect size, i.e., the expected difference between the two groups—conventionally, 0.2 = a small effect, 0.5 a medium effect, and 0.8 a large effect; and standard deviation (σ), i.e., the variability in the data

### 2.2. Formula for Sample Size

For a comparison of two means (e.g., urban vs. rural groups), the sample size for each group can be calculated using the following formula:

$$n=\frac{2\left(\sigma^{2}\right){\left(Z_{\alpha /2}+Z_{\beta }\right)}^{2}}{{\left(\mu_{1}-\mu_{2}\right)}^{2}}$$

where:

*n*: Sample size per group.

*σ*: Standard deviation.

*Z_α_*_/2_: Z-value corresponding to the significance level (for *α* = 0.05, *Z_α_*_/2_ = 1.96).

*Z_β_*: Z-value corresponding to the desired power (for 80% power, *Z_β_* = 0.84).

*μ*_1_ − *μ*_2_: The expected difference in means between the two groups (effect size).

We should have 63 participants per group to detect a 5-unit difference with 80% power and a 0.05 significance level. The eligibility criteria included individuals aged 65 or over, with no serious cardiovascular pathology or chronic musculoskeletal symptoms (physical performance test) that made walking difficult, with satisfactory cognitive analysis results for the exercise (Mini Mental State Exam, Folstein), exclusion criteria were: very active individuals (IPAQ assessment), individuals with falls risks, as well as individuals with poor adherence to the project.

To access this information, we performed the following tests.

1. Physical performance test. This is a standardized test designed to evaluate physical function and performance, especially in elderly individuals. It assesses the ability to perform tasks that simulate daily activities and measures overall functional status. The PPT is particularly useful for identifying limitations in mobility and physical function, which can be critical in determining the risk of disability or the need for rehabilitation. The PPT consists of 7 or 9 tasks (depending on the version) that reflect common daily activities. The tasks are scored based on the time taken or the quality of performance. Each task is typically scored from 0 to 4, with a total score range depending on the version used.
2. Mini Mental State Examination (MMSE), also known as the Folstein test. This is a widely used cognitive assessment tool designed to screen for cognitive impairment and evaluate cognitive function. It is often used in clinical settings to assess memory, orientation, attention, language, and visuospatial abilities, particularly in elderly individuals, and it helps in diagnosing conditions such as dementia, including Alzheimer’s disease. The MMSE is a short, structured questionnaire that typically takes 5–10 min to administer. It consists of 30 points divided into different categories that assess various cognitive functions.
3. International Physical Activity Questionnaire (IPAQ). This is a standardized, self-reporting tool designed to measure physical activity levels in adults. It assesses the frequency and duration of physical activity across different domains, such as work, transportation, household tasks, and leisure-time activities. The IPAQ is widely used in both research and clinical settings to evaluate physical activity and sedentary behavior, providing data that can be compared across populations globally.
4. Morse Fall Scale. This is a standardized method to evaluate the likelihood of a fall by assessing several risk factors. Each risk factor is assigned a point value, and the total score determines the patient’s fall risk category. Based on the score, preventive measures can be implemented to reduce the likelihood of falls.

The MFS consists of six variables that are commonly associated with the risk of falling. Each variable is assigned a point value based on the level of risk.

### 2.3. Procedures and Materials

#### 2.3.1. Participants and Procedures

After the project was approved by the ISAVE’s ethics committee (050523) and registered (ACTRN12624001481561), patients were called through contacts with the Landscape Laboratory, the municipality of Guimarães, and Tempo Livre.

Firstly, the participants signed a declaration of informed consent to take part in the study, and after that, data concerning sample characterization were collected.

On the first assessment day, parameters such as heart rate (HR), blood pressure (BP), peripheral capillary oxygen saturation (SpO_2_), analysis of physical capacity (IPAQ, physical performance test), risk of falls (Morse Fall Scale), Mini Mental State Examination, and perception of accessibility (Perception of Pedestrian Accessibility Scale for adults over 65) were collected. Data concerning heart rate, blood pressure, saturation, physical performance, and neurological condition were again measured on the last day of the study.

The Perception of Pedestrian Accessibility Scale for adults over 65 is a tool designed to evaluate how elderly individuals perceive the accessibility and ease of use of pedestrian environments in their daily lives. This scale helps assess factors related to the walkability of an area, including physical and environmental features that may influence an older adult’s ability to move safely and comfortably on foot.

On the first assessment day, participants attended an evaluation session in a quiet, reserved room at the study site to ensure confidentiality and comfort. The session was conducted individually to maintain privacy and minimize external influences.

All questionnaires were administered in Portuguese, the participants’ native language, and provided in paper format. Each participant received clear oral and written instructions on how to fill in the questionnaires. Trained researchers were present to clarify doubts or assist participants when necessary, while ensuring responses remained unbiased.

The questionnaires were completed in a quiet, well-lit room with comfortable seating to provide a distraction-free environment. On average, it took 45 to 60 min to complete all the questionnaires, with small breaks offered if needed to accommodate participants’ comfort. All questionnaires were filled in individually, without the presence of other participants, and handed to the researcher immediately upon completion. After completing the questionnaires, participants were randomly divided into two groups using the software at www.randomizer.org (version 4.0, accessed on 22 May 2023) to ensure unbiased allocation.

Data concerning heart rate, blood pressure, oxygen saturation, physical performance, and neurological condition were measured again on the last day of the study.

#### 2.3.2. The Two Routes—Procedures

**Figure 1:**
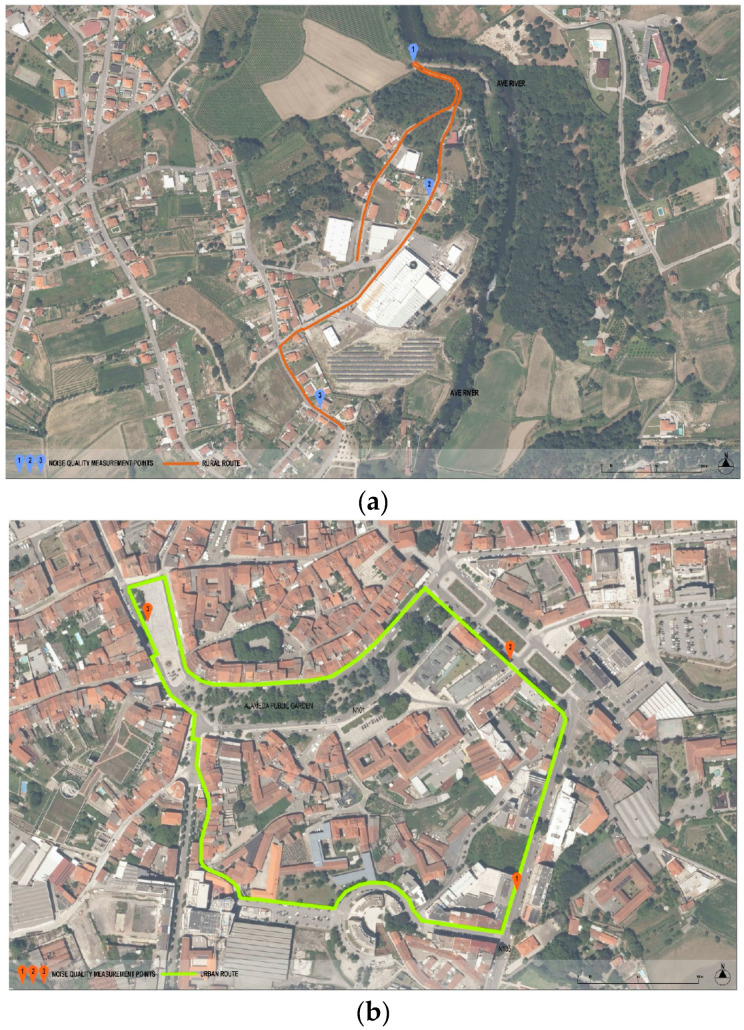
Rural (**a**) and urban (**b**) routes chosen for the study, along with the noise measurement points.

Regarding the environmental characteristics of the two selected areas (Figure 1), temperature conditions were recorded every 300 m (thermometer). To obtain the environmental noise data, a tripod, a class 1 acoustic calibrator, and a class 1 integrating sound level meter in compliance with recommendation 88 of the International Organization for Legal Metrology (OIML) and the international standard IEC 61672 [17] were used. The sound level meter used was a CESVA model SC310, approved by the ISQ. For the ambient noise measurements, the tripod was used at a height of 1.5 m off the ground, with the microphone directed toward the main noise source. To evaluate the equivalent continuous sound level indicator (Leq), the statistical indicators L_5_ (5% of the time, the noise level is above this level, which usually represents sporadic or intermittent annoying peaks of noise) and L_95_ (95% of the time, the noise level is above this level, which usually represents the background or ambient level of a sound environment) were measured, and the presence or absence of impulsive characteristics was measured using the integration speeds “fast” and “impulse” simultaneously.

Three ambient noise measurement points were established along each pedestrian route to assess the experience of walking in rural and urban areas. Different characteristics, such as sidewalk type and surroundings, were considered to better represent each route. At each point, two continuous 10 min noise measurements were conducted, resulting in a total of 60 min of measurement on each route. The measurements were carried out on 25 May 2023, from 3:30 p.m. to 8:30 p.m.

Air quality was obtained from data provided by the air quality monitoring station of Guimarães and the air quality chart of the municipality of Guimarães.

#### 2.3.3. Task

Participants were divided into two groups—one that performed the task along the urban route and one that performed the task along the rural route—with each route being at least 1.6 km long. In each group, they were asked to walk twice a week for six weeks at a speed they considered comfortable, and were always accompanied by the researcher [18]. All participants were duly insured against personal injury. Heart rate, systolic blood pressure, and diastolic blood pressure were measured before and after the walk using portable electronic sphygmomanometers. Measurements were performed in a relaxed sitting position, and the instrument was placed at heart level.

### 2.4. Ethics

The study was submitted for approval to the ISAVE ethics council. Data collection took place between April and May 2023.

All participants were informed about the objectives and procedures involved and had to declare their acceptance of participation in the study (informed consent). They could withdraw at any time as per the Declaration of Helsinki. Participants were also assured of data anonymity and confidentiality, and were guaranteed that it would not be used for purposes other than this scientific research. To guarantee anonymity, each participant was given a numerical code, which was not identified in any of the instruments used, and the consent form was separated from the other documents.

### 2.5. Statistical Procedures

The data were analyzed using the statistical analysis software IBM SPSS^®^ 29 (IBM, Armonk, NY, USA) for Microsoft Windows.

Firstly, the normality of distribution of the variables was assessed using the Shapiro–Wilk test, and as we found that the variables did not follow a normal distribution, we decided to use non-parametric tests for data analysis. The sample under study was characterized using descriptive statistics: mean (standard deviation), median, and interquartile range (IQR).

For statistical analysis, with a significance level of 5%, the *t* test was used to compare the anthropometric variables of the two groups under study, and the Wilcoxon test was used to compare the variables under study at both times. The Mann–Whitney test was used to compare variables at the two sites at both times.

## 3. Results

This study involved 24 individuals divided into two groups.

Both groups were firstly assessed in terms of the Perception of Pedestrian Accessibility Scale for adults over 65 (PAP) scale (*p* < 0.081), with the highest value found being 35 (out of a maximum of 52), which suggests a reasonable perception of pedestrian accessibility in the regions under study and Morse Fall Scale (*p* = 1), with no statistically significant differences found.

After analysis of both samples using the *t* test, we found that there were statistically significant differences between group 1 (3.08 (0.5)), considered active, and group 2 (2.4 (0.7)), considered insufficiently active, in terms of IPAQ data (*p* < 0.009).

Subsequently, the two moments (initial and final) were compared in each group. The results are shown in Table 1 and Table 2.

The table above shows that statistically significant differences were found for weight, SpO_2_, and the Mini Mental State Examination.

The table above shows statistically significant differences for SpO_2_ and PPT.

We also compared the two groups at the two assessment times (initial and final) and found no statistically significant differences. The results are shown in Table 3 and Table 4.

The variables of noise and air quality were also assessed. Three points were defined along the urban route, with two measurements lasting 10 min each for a total of 60 min of evaluation (Table 5 and Table 6).

No impulsive characteristics were observed. However, the average energy levels of the equivalent continuous sound level indicator (Leq) exceeded the Lden limit of 71 dB(A) at the three points along the urban route.

The evaluation was carried out during the day, but cannot be directly compared with the Lden limit values, as the measurement would have to be conducted over 24 h, covering day, dusk, and night, as stated in the Practical Guide for Ambient Noise Measurements/RGR/NP ISO 1996 [4]. Nonetheless, it still provides insight into the specific situation, which is the objective of this study.

**Figure 2:**
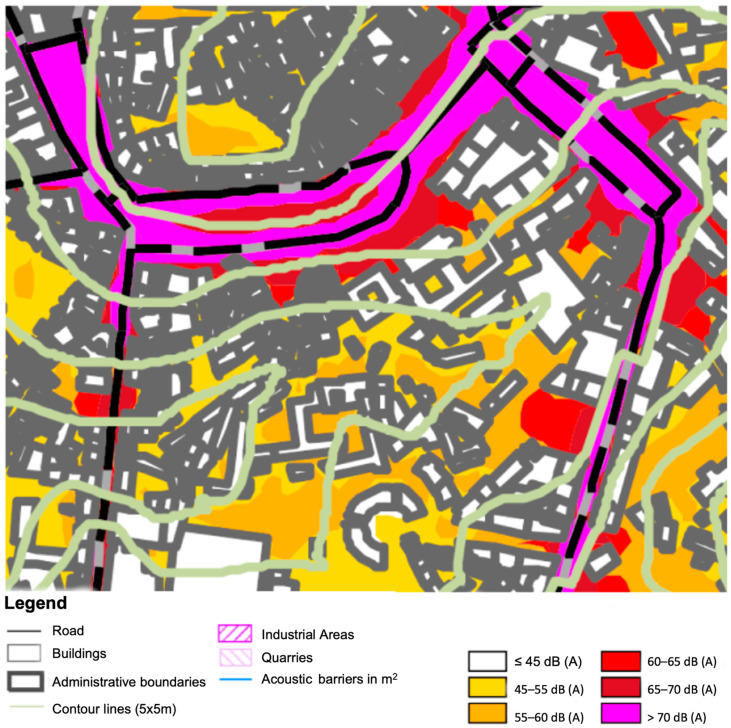
Excerpt from the Guimarães Noise Map—Lden B Chart (Limp.AR project).

Comparison with the Guimarães Noise Map reveals that at the three points, the modeled values exceeded 70 dB(A), very close to the measured values. This area of the city experiences significant acoustic conflict, with overexposure values ranging from 5 dB(A) to 10 dB(A) (Figure 2).

Three points were defined along the rural route, where two measurements lasting 10 min each were taken, for a total of 60 min of assessment.

No impulsive noise characteristics were observed during this period, and the energy averages of the continuous sound level indicator equivalent (Leq) did not exceed 61 dB(A), with the average over the course being 58 dB(A). Comparing the data with the modeling carried out on the Guimarães Noise Map, the average noise in this area over the 24 h of the day is lower than 55 dB(A), so there is no acoustic conflict.

It is important to note that during the experimental procedure and according to the meteorological stations in Guimarães, the average temperature recorded during the study was 14.9 °C, which is a normal temperature for the time of year. The average annual temperature is 10.4 °C.

Regarding air quality, the city of Guimarães has an air quality map that utilizes an index called CityAir. This index considers the concentration of the pollutants PM_10_ (fraction of particulates with an aerodynamic diameter smaller than 10 μm), NO_2_ (nitrogen dioxide) and SO_2_ (sulfur dioxide) to classify air quality on a scale from very poor to very good. Despite heavy traffic, the urban area studied has good to very good air quality, while the rural area has very good air quality.

Additionally, data from the air quality monitoring station located in Azurém, Guimarães were considered. This station uses the QualAR index, which classifies air quality from “bad” to “very good” and is based on reference values from the WHO in 2019. On 25 May 2023, only concentration data for pollutants NO_2_ and PM_10_ were available (Table 7). From these, it was found that air quality during the noise measurement period (3:30 p.m. to 8:30 p.m.) varied from good to very good.

Also, according to the 2023 Green City Accord report, Guimarães follows WHO guidelines, setting targets of PM_2.5_ < 4 µg/m^3^ by 2030. Additionally, the city has committed to a local interim target of NO_2_ of 20 µg/m^3^.

## 4. Discussion

As stated by Fonseca et al. [5], green areas tend to promote psychological and physiological benefits for people’s health and well-being, such as making them feel better, evoking a sense of relaxation, reducing stress and anxiety, and helping decrease depression and negative feelings. Despite this, the relationship between these areas and health/well-being is not entirely clear and should be examined, preferably through physiological and psychological measurements.

Along these lines, this study aimed to understand the influence of two areas, one rural and one urban, on multiple physiological and functional variables, as well as to assess the perception of individuals regarding the ease or otherwise of using these areas.

There is a relevant importance of vegetation for human well-being, as well as the presence of places such as city parks to encourage socialization and exercise. One of our goals was to verify if our sample was aware of the existence of this kind of facility. For that, we used the Perception of Pedestrian Accessibility Scale for adults over 65 (PAP), and we found they had reasonable perceptions of pedestrian accessibility in the regions under study. The population over 65 already has physical and cognitive transformations and a potential reduction in their economic resources due to retirement. For these people, the evolving area is truly important [19]. Recognizing these areas and the ability to use them is fundamental to keeping these people active. Concerning physical activity, one frequent risk in this age range is the risk of falls. Before our intervention, we also evaluated the fall risk with the Morse Fall Scale. None of our individuals presented a risk of falling, so the option of walking was feasible.

Hansen Li et al. [18] looked at how walking in urban walking interfered with the physiological and psychological characteristics of the participants. They found positive results regarding blood pressure and psychological state when analyzing walking in green areas, unlike walking in urban areas. Exposure to green spaces is associated with a decrease in heart rate and an increase in heart rate variability, indicating improved cardiovascular health. This effect is attributed to stress relief and reduced exposure to air pollution and noise in green environments. Also, green spaces can lead to lower blood pressure, particularly systolic blood pressure, during and after exposure. This effect is observed in both short-term visits and regular physical activities in green environments. Enhanced oxygen saturation may be inferred from improved overall cardiovascular function and reduced stress, which are induced by increased physical activity [20,21]. Additionally, we must highlight that pulse oximeters can have a margin of error of approximately ±2%. For a healthy individual, a change from 98% to 97% peripheral capillary oxygen saturation is negligible. Although SpO_2_ showed statistically significant differences, these lack clinical relevance, as they remain within normal limits and may reflect natural variability or equipment error margins. Future studies should monitor these variables in real time along the routes to obtain more accurate data. In the present study, we found that walking in urban areas improved scores on the Mini Mental State Examination, but the same was not true for the group that walked in rural areas. One possible explanation could be the stimulation that group 1 was subjected to, because the pedestrian circuit was urban with a lot of traffic, stores, and so on. Another factor could be related to socializing, since more and more people are in urban areas, and due to the pace of life, the elderly have become more isolated, thus reducing their cognitive skills [22].

Regarding the participants’ level of physical activity, those in group 1 were more active than those in group 2, in line with Wang and Wilcox [23,24], who pointed out that a lack of physical activity and sedentary behavior were also observed in rural areas, especially among older adults, in line with the results mentioned above. Regis [25] points out that the different characteristics of urban and rural areas, such as population density, safety, and access to facilities for practicing physical activity, can influence the level of physical activity and the behavior of the population. On the other hand, Vaz et al. [26] identified that older individuals from rural areas tend to have higher levels of physical fitness compared to urban areas.

However, this fact does not corroborate our results. Even though we had a sample of older women, we found a lower level of sedentary lifestyle in the urban population.

After six weeks of the study, group 2 showed improvements in PPT, unlike group 1. It is therefore possible that the twice-weekly practice may have positively influenced physical performance on the multiple domains of physical function, increasing capacity for activities of daily living, in line with Porto et al. [27]. It is important to highlight that despite excluding severe health conditions, body mass index variations may have contributed to differences in performance outcomes, such as gait speed and endurance. This should be considered when interpreting the PPT results.

Regarding cardiorespiratory variables, only saturation suffered significant changes in both groups. On the one hand, in the urban group, we could explain the slight drop in saturation by the fact that it was an urban route, but the data obtained on air quality do not allow us to draw such a conclusion, since in the municipality of Guimarães, the air quality is classified as good (according to CityAir-Index, 97.3% of the population is exposed to good/very good air quality indices).

On the other hand, in group 2, walking in the rural setting, we would not expect to find a similar result. It is important to add that we do not have real-time air quality measurements in rural areas, so we cannot correlate these results with air quality.

Once again, if the air quality in urban areas is not dangerous, we believe that it would be pertinent to replicate the study by monitoring the saturation data along the entire route to obtain more precise data to understand whether or not the environment influences the cardiorespiratory variables analyzed by Stieb et al. [28].

Regular physical exercise is a key factor in weight loss. We found that group 1 lost a statistically significant amount of weight. Despite the results showing that they were the most active group, the practice of walking still seems to have had an impact on their weight and not on their functional capacity, since we found no differences in the physical performance test, unlike group 2.

According to Ranzani et al. [29] and Mariosa [30], the prevalence of hypertension increases over time, the rate of change is higher in rural areas than in urban areas, and the risk of prevalence of hypertension may be associated with determining environmental factors, which include air pollution, noise pollution, and access to healthy food. Most studies have reported the benefits of exposure to green areas on blood pressure.

A higher normalized difference vegetation index (NDVI) is significantly associated with lower levels of systolic blood pressure.

In addition, this same index in different zones and the proportion of green spaces are also significantly associated with lower probabilities of hypertension according to Zhao [31]. However, the results obtained do not allow us to corroborate the studies described above, since we did not find significant results in the cardiac variables analyzed. As expected, the short duration of the study may explain the absence of statistically significant differences in several physiological variables between the groups. Any differences observed should be interpreted cautiously, as the impact of the environment may require longer-term exposure to be fully understood.

As far as noise is concerned, we found the expected differences between the two environments when measuring noise, and in urban environments, the values exceeded the advisable.

The predominance of women may influence the generalizability of the results. However, this also provides a significant opportunity to gather important insights into health and wellness issues specifically relevant to women. The findings could serve as a foundation for further research focused on gender-specific responses to environmental exposure and physical activity. Another limitation is the absence of equipment to monitor cardiorespiratory variables along the route. The small sample is a limitation of the study, which may reduce the ability to detect statistically significant differences between the groups, particularly given the relatively short duration of the intervention. Therefore, the methodology of the study could be improved, as well as applied to a larger and more representative sample, to understand how the practice of exercise can be influenced by the environment and its inherent implications for physiotherapy results.

It is important to highlight that during the study, the only operational air quality station in the city provided data only regarding the concentration of NO_2_ and PM_10_, and it is in the city center. In future studies, a larger sample of people with varied demographic characteristics should be considered, as well as the collection of specific environmental data from both routes, namely, using mobile air quality monitoring stations (PM10, PM 2.5 and TSP: total suspended particulate matter) and noise. The measurement of fine particles (PM2.5) is of particular interest, as these are strongly associated with health risks such as heart disease and asthma, thereby increasing the reliability of the results. Only in this way will it be possible to carry out a more in-depth study of the relationships between environmental aspects and the various demographic variables.

In future research, we believe we should consider incorporating real-time environmental data, such as PM2.5 and PM10, to enhance the accuracy of the analyses. Additionally, it is crucial to investigate the impact of environmental variables independently in subsequent studies to better understand their specific effects, for example, integrating noise and air quality in a follow-up study and exploring their role.

## 5. Conclusions

The practice of therapeutic exercise in the open air could be closer to the reality of each user. The results of this study seem to indicate that the practice of walking in urban and rural environments can have a positive impact on the health of elderly individuals. However, further research with larger and more diverse samples is needed to confirm these results. Despite this, it is important to highlight that the benefits of outdoor activities can be compromised by poor environmental quality. As mentioned earlier, noise and air quality can negatively impact public health, especially among vulnerable groups such as the elderly. In the study conducted, both urban and rural routes were found to have good air quality. However, the same cannot be said for noise, as the urban route presented values that exceeded those recommended by the WHO. Future studies should assess the health impact on elderly individuals being exposed to noise during exercise along these two routes. This study underscores the crucial role of enhancing green infrastructure in boosting ecosystem services and amplifying the health benefits of accessible green spaces. Strengthening strategies that foster rural–urban connections through the active involvement of local administrations and civil society can significantly contribute to more effective urban planning.

This study emphasizes the need for research and development to guide city policymakers in integrating green spaces, in line with the WHO’s recommendation that everyone should live within 300 m of green areas. It underscores the crucial role of enhancing green infrastructure in boosting ecosystem services and amplifying the health benefits of accessible green spaces. Strengthening strategies that foster rural–urban connections through the active involvement of local administrations and civil society can significantly contribute to more effective urban planning.

## Figures and Tables

**Table 1:** Comparison between the two moments (Wilcoxon test) regarding weight (kg), SpO_2_ (%), heart rate (bpm), systolic blood pressure (mmHg), diastolic blood pressure (mmHg), Mini Mental State Examination, and physical performance test. Median and interquartile range (IQR) values for group 1.

Variables	Median (IQR)	*p*-Value
Weight	68.8 (12.38)	0.021 *
	67.3 (13.75)	
SpO_2_	98.0 (0.75)	0.033 *
	97.0 (1)	
HR	80.5 (19.75)	0.624
	77.0 (11.5)	
SBP	133.0 (40.25)	0.241
	130.0 (12.25)	
DBP	76.5 (16.0)	0.689
	81.0 (1.7)	
Mini Mental State Examination	27.5 (2.5)	0.041 *
	29.0 (2.0)	
PPT	25.5 (1.75)	0.751
	25.0 (1.0)	

SpO_2_: peripheral capillary oxygen saturation; HR: heart rate; SBP: systolic blood pressure; DBP: diastolic blood pressure; PPT: physical performance test. * Statistically significant (*p* < 0.05).

**Table 2:** Comparison between the two moments (Wilcoxon test) for weight (kg), SpO_2_ (%), Heart Rate (bpm), systolic blood pressure (mmHg), diastolic blood pressure (mmHg), Mini Mental State Examination, and physical performance test. Median and interquartile range (IQR) values for group 2.

Variables	Median (IQR)	*p*-Value
Weight	70.85 (11.33)	0.142
	69.5 (11.2)	
SpO_2_	98.0 (1.0)	0.008 *
	97.0 (1)	
HR	73.0 (13.75)	0.683
	76.0 (9.75)	
SBP	146.5 (34.0)	0.625
	131.6 (21.25)	
DBP	80.0 (11.75)	0.409
	81.0 (11.0)	
Mini Mental State Examination	28.5 (1.0)	0.671
	28.0 (1.75)	
PPT	25.0 (1.0)	0.041 *
	28.5 (2.5)	

SpO_2_: peripheral capillary oxygen saturation; HR: heart rate; SBP: systolic blood pressure; DBP: diastolic blood pressure; PPT: physical performance test. * Statistically significant (*p* < 0.05).

**Table 3:** Comparison between the two groups (Mann–Whitney test) for weight (kg), SpO_2_ (%), heart rate (bpm), systolic blood pressure (mmHg), diastolic blood pressure (mmHg), Mini Mental State Examination, and physical performance test. Median values for moment 1.

Variables	Median (IQR)	*p*-Value
Weight	69.1(11.7)	0.795
SpO_2_	98.0 (1.0)	0.596
HR	75.0 (14.0)	0.862
SBP	140.5 (34.5)	0.525
DBP	77.0 (14.0)	0.686
Mini Mental State Examination	28.0 (2.0)	0.104
PPT	25.0 (1.75)	0.088

SpO_2_: peripheral capillary oxygen saturation; HR: heart rate; SBP: systolic blood pressure; DBP: diastolic blood pressure; PPT: physical performance test.

**Table 4:** Comparison between the two groups (Mann–Whitney test) for weight (kg), SpO_2_ (%), HR (bpm), SBP (mmHg), DBP (mmHg), Mini Mental State Examination, and PPT. Median values for moment 2.

Variables	Median (IQR)	*p*-Value
Weight	68.0 (12.4)	0.583
SpO_2_	97.0 (2.25)	0.187
HR	77.0 (11.5)	0.706
SBP	130.5 (16.25)	0.214
DBP	81.0 (11.75)	0.664
Mini Mental State Examination	29.0 (1.75)	0.341
PPT	25.0 (1.0)	0.713

SpO_2_: peripheral capillary oxygen saturation; HR: heart rate; SBP: systolic blood pressure; DBP: diastolic blood pressure; PPT: physical performance test.

**Table 5:** Noise along urban route.

Point	Leq (dBA)	L_5_ (dBA)	L_95_ (dBA)	Impulsive Noise
	Average	Average	Average	Yes/No
1	68.9	73.5	60.5	no
2	67.2	72.1	60.3	no
3	74.1	82.2	57.2	no
Urban route	71.1	78.4	59.6	-

**Table 6:** Noise along rural route.

Point	Leq (dBA)	L_5_ (dBA)	L_95_ (dBA)	Impulsive Noise
	Average	Average	Average	Yes/No
1	50.6	53.6	43.4	no
2	54.8	62.5	44.0	no
3	61.2	65.5	41.5	no
Rural route	57.6	62.7	43.1	-

**Table 7:** Data source: QualAR—APAmbiente (https://qualar.apambiente.pt/indices (accessed on 16 october 2024)).

Air Monitoring Station: Cón. Dr. Manuel Faria. Azurém		
Station Type:	Urban	
Date:	25 May 2023	
Pollutant:	NO_2_	PM_10_
Hour (UTC)	Average (μg/m^3^)	Average (μg/m^3^)
0	30.0	16.0
1	27.0	14.0
2	22.0	15.0
3	16.1	14.0
4	18.1	8.0
5	14.1	-
6	21.0	-
7	30.0	-
8	33.0	-
9	28.1	-
10	19.1	11.0
11	24.1	13.0
12	23.0	13.1
13	29.0	13.1
14	29.0	23.1
15	32.1	21.0
16	42.0	23.0
17	51.0	27.0
18	52.0	23.1
19	43.0	21.0
20	53.0	22.0
21	45.1	19.0
22	33.0	19.0
23	25.0	10.0

## Data Availability

The original contributions presented in this study are included in the article. Further inquiries can be directed to the corresponding author.
